# The Role of Single-Nucleotide Polymorphisms in Pituitary Adenomas Tumorigenesis

**DOI:** 10.3390/cancers11121977

**Published:** 2019-12-09

**Authors:** Sumedh S. Shah, Manish K. Aghi

**Affiliations:** Department of Neurological Surgery, University of California, San Francisco, CA 94143, USA; sumedhss@med.miami.edu

**Keywords:** pituitary adenoma, single-nucleotide polymorphism, tumorigenesis

## Abstract

Pituitary adenomas (PAs) are among the most common intracranial neoplasms, but despite their histologically benign nature, these tumors sometimes grow large enough to cause symptoms of mass effect such as vision loss, headaches, or hypopituitarism. When they get this large, surgery will unfortunately not be curative and, other than prolactinomas, medical options are limited, and radiation has variable efficacy in controlling growth. Understanding the genetic perturbations, such as single nucleotide polymorphisms (SNPs), that promote the formation or growth of functional and nonfunctional PAs is important because such genetic insights could improve the diagnosis and subsequent classification of PAs as well as unlock potential therapeutic targets outside contemporary standard of care. While there have been great strides in the research of SNPs as drivers of PA formation and maintenance, a comprehensive discussion of these genetic mutations has not been undertaken. In the present article, and with the goal of providing scientists and clinicians a central review, we sought to summarize the current literature on SNPs and their relationship to PA formation. Across multiple tumor types, such as nonfunctioning PAs, prolactinomas, corticotroph adenomas, somatotroph adenomas, thyrotropic adenomas, and gonadotroph adenomas, SNPs in cell surface receptors implicated in proliferation can be appreciated. Polymorphisms found in tumor suppressors and cell cycle regulators have also been identified, such as p53 SNPs in nonfunctioning PAs or cyclin D1 in prolactinomas. While the translational relevance of SNPs in the formation of PAs is still in the early stages, the use of wide-scale genomic analysis to identify patients at risk for developing PAs could yield therapeutic benefit in the future.

## 1. Introduction

Pituitary adenomas (PAs) are benign neoplasms of the pituitary gland that represent the second most common intracranial tumor with an estimated rate of 3.91–3.97 persons per 100,000 [1,2]. The incidence of PAs has been steadily increasing, possibly secondary to an aging population coupled with increased use of surveillance imaging in patients with nonspecific neurologic complaints [3,4]. General classification schema delineate PAs based on size (i.e., microadenoma, <1 cm, versus macroadenoma, ≥1 cm), immunohistochemistry staining, secretory endocrine status (i.e., functional versus nonfunctional), and primary cell of origin with type of hormone secreted—in cases of functional PAs [5,6]. Functional tumors, with transcriptional factor subclassification, include lactotroph (prolactin (PRL)-secreting, Pit-1+), somatotroph (growth hormone (GH)-secreting, Pit-1+), corticotroph (adrenocorticotropin (ACTH)-secreting, Tpit+), gonadotroph (SF-1, ER, GATA-2+), thyrotroph (Pit-1, GATA-2, TEF+), and plurihormonal subtypes [7,8]. Recently, null cell adenomas have been separately classified as a rare type of tumor as having no evidence of lineage differentiation by either transcription factors or hormonal status [6].

Clinical manifestation of these neoplasms ranges from headache or neurological deficit due to compression of adjacent structures, such as bitemporal hemianopsia in cases of chiasmal involvement, to hormonal disturbances caused by functional PAs [9]. Management of PAs is dependent on cellular subtype, with prolactinomas responding vigorously to primary medical dopaminergic therapies [9]. For other subtypes, gross total surgical resection via microscopic or endoscopic transsphenoidal approaches is standard of care [10,11,12]. Adjuvant medical modalities can also be utilized as surgical adjuncts for some functional PAs for subtotally resected primary or recurrent tumors, with the latter consideration important because recurrence can be as high as 10%–30% for some types of functional PAs, even in cases of gross total resection [13]. For example, surgical management of somatotroph tumors can be augmented with somatostatin receptor ligands or growth hormone receptor antagonists [9]. However, regardless of optimal therapeutic approaches and the overall benign nature of these tumors, PAs can still lead to significant comorbidities and decreased quality of life (i.e., Cushing’s syndrome in ACTH-secreting tumors; acromegaly in GH-secreting tumors) [14,15]. In addition to the mass effects of adenomas or aberrant pituitary hormone production, PAs can invade laterally into the cavernous sinus, which then results in residual tumor requiring multimodal approaches to achieve tumor control and/or biochemical remission—particularly in patients with hormonal hypersecretion [16].

Thus, it remains imperative to improve our overall holistic understanding of biochemical, molecular, genetic, and epigenetic drivers of PA tumorigenesis as this may yield novel therapeutic targets and improve the prognosis of difficult-to-treat tumors. A recent literature review by Faltermeier et al. summarizing the molecular biology of PAs suggested that their pathogenesis is multifactorial, with certain somatic mutations, alterations in gene transcription, and epigenetic changes interacting to promote tumorigenesis [17]. Other studies have focused on differential protein expression or epigenetic profiles that could give rise to some PAs [17]. Beyond these areas of investigation, several studies have investigated the role of single-nucleotide polymorphisms (SNPs) in PAs. Here, we comprehensively analyze the relationships between SNPs and PA tumorigenesis—stratified by relevant histological subtypes—and highlight the translational relevance of SNPs in PAs.

## 2. Single-Nucleotide Polymorphisms (SNPs) in Pituitary Adenomas by Tumor Type

### 2.1. Definitions–Mutation vs. Polymorphism

Sequence variations exist at defined positions within the genome and are responsible for individual phenotypic characteristics. Single nucleotide polymporphisms (SNPs) are these naturally occurring substitutions of a single nucleotide in specific sequences of the germline DNA where each resultant variation is present to an appreciable degree within a population (detectable in greater than 1% of a population). Certain population groups based on variables such as ethnicity or geography can exhibit similar SNP profiles, which, in turn, can be critical to neoplasm formation. Thus, SNP analysis can be particularly beneficial as a predictive indicator for cancer risk in certain populations where that polymorphism is common. This is in contrast to a mutation, which is any change in the germline or somatic cell DNA sequence that deviates from what is considered normal (detectable in less than 1% of a population).

A common methodology used to study SNPs is the genome-wide association study (GWAS). This tool is an observational study of a genome-wide set of genetic variants in different individuals to determine if any variant is associated with a trait. Evolutionarily, SNPs are advantageous in order to maintain a diverse genetic pool, however, SNPs also relate to propensity towards development of complex disease states, including cancer, and can be implicated in tumor progression or response to therapy [18,19]. Given the established role of SNPs in various tumors, investigation into their relationship to PA formation and maintenance is warranted.

### 2.2. Brief Background on Mutations in Pituitary Adenomas

Mutational events as drivers for PA formation have been well documented in contemporary literature. Thus, a brief discussion regarding key drivers is warranted, particularly since the subject of this article is specifically on SNPs. As mentioned earlier, Faltermeier et al. produced a comprehensive analysis of the molecular biology of pituitary tumorigenesis by tumor type [17]. Both germline and somatic mutations have been associated with formation of some PAs. Germline mutations in the *MEN1* tumor-suppressor gene have been found in PRL-secreting tumors. Somatic mutations in *BRAF* and ubiquitin specific peptidase (USP) genes, specifically USP8 and USP48, have been associated with corticotroph adenomas. Combinations of germline mutations (aryl hydrocarbon receptor interacting gene (*AIP*), protein kinase cAMP-dependent type 1 regulatory subunit alpha (*PRKAR1A*), G-coupled protein receptors) and somatic mutations (G-protein alpha subunit 1 (*GNAS1*)) are associated with somatotroph formation [17]. These clear examples of driver mutations in some pituitary tumors are contrasted by the lack of identified driver mutations in thyrotroph, gonadotroph, and null cell adenomas.

### 2.3. SNPs Found in PAs in General (Functional and Nonfunctional)

While the following discussion will focus on SNPs per PA functional and secretory status, review of the literature reveals additional pertinent polymorphisms and their relationship to PAs in general. Important sets of proteins of interest in PA invasiveness worth including are the metalloproteinases (MMPs) and their associated SNPs. MMPs can promote invasion by degradation of collagen found in basement membranes of tissues. Thus, PAs exhibiting high MMP activity can be associated with aggressive tumor phenotype. Zlatkute et al. (2017) discussed the role of *MMP-1* polymorphisms on the development of PAs by analyzing genetic profiles of 100 patients with PA and comparing them with healthy controls [20]. They found that the polymorphism in the MMP-1 gene 1G/1G was more frequent in the invasive group of PAs and in the more active tumors than in control PAs (*p* = 0.044), suggesting that this polymorphism may play a role in PA development [20]. This was in contrast to a previous paper by Atlas et al. (2010), in which they found that *MMP-1* 2G/2G alleles were more common in the invasive PAs than 1G/1G variants, but that allelic pattern was not statistically significant compared with control [21]. Beyond *MMP-1*, the rs3918242 C > T SNP in the *MMP-9* gene was suggested to play an important role in the development of PAs, a particularly interesting result as studies have suggested that higher secretion of MMP-9 is observed in invasive PAs compared with noninvasive PAs [22,23].

### 2.4. SNPs in Nonfunctional Pituitary Adenomas

Nonfunctional PAs (NFPAs) are a common subtype of PA, accounting for roughly 25% of all PAs, but are not associated with hormonal hypersecretion [24,25]. Progression-free survival and recurrence rates are favorable amongst these tumors [24,26]. Arising from adenohypophyseal cells, these masses present either incidentally on imaging or via symptoms secondary to cranial mass such as headache, pituitary dysfunction, or visual disturbances. Therapy in way of hormonal replacement and/or surgical management via transsphenoidal approach is directed at correcting hypopituitarism or eliminating mass effect on adjacent neural structures, respectively [27]. Despite the availability of therapeutic modalities to treat NFPAs, the genetic mechanisms that lead to the initiation of NFPAs remain incompletely understood, particularly as pertaining to SNPs.

Recent investigation into the relationship between SNPs and NFPAs has revealed SNPs over-represented in NFPA patients relative to the general population in genes regulating tumor suppressors, cell cycle control proteins, and cell surface receptors. Yagnik et al. (2017) investigated the role of p53 polymorphisms in the development of NFPAs. Their study found that the polymorphism rs1042522 C > G in codon 72 of exon 4 of the *TP53* gene, whose C variant produces a proline and is more common in most ethnicities, has a G variant producing an arginine in around 80% of NFPAs [28]. The tumor suppressor protein, p53, functions to conserve genomic stability and, in cases of irreparable damage, can guide cells towards cell cycle arrest via interaction with p21 mediator proteins or towards apoptosis [29]. In NFPA, the rs1042522 G variant polymorphism affects not only p53 but can also lower p21 protein expression while simultaneously raising vascular epithelial growth factor (VEGF) levels. The clinical relevance of this result suggests that NFPA cells harboring this SNP promote adenoma growth by undergoing increased cellular proliferation coupled with increased vascularity. As such, these present almost a decade earlier with symptomatic NFPAs. Interestingly, when wild-type cultured NFPA cells were transfected with the rs1042522 G variant, a concomitant decrease in p21 was noted along with increased cellular proliferation, suggesting that this *TP53* SNP influenced NFPA growth [28].

Not only do SNPs in tumor suppressor genes seem to correlate with NFPA development and proliferative capability, but polymorphisms in growth factor receptors have also been described as being associated with NFPAs. In a case-controlled study by Zhu et al. (2018), the authors found that the rs2981582 AA genotype polymorphism in the fibroblast growth factor receptor 2 (*FGFR2*) gene was associated with NFPA within the Chinese Han population [30]. FGFR2 is of the tyrosine kinase receptors, and so aberrant activity of FGFR2 can mediate tumorigenesis by activation of mitogenic and pro-survival signals, including MAPK, AKT, and RAS, as well as by promoting angiogenesis and invasion. In addition to finding that SNPs in FGFR2 lead to increased tumor risk, the authors found that the AA allele significantly correlated with NFPA morbidity, even after adjusting for various demographic factors such as sex, age, body mass index, smoking status, and alcohol consumption. Ultimately, while focusing on one polymorphism cannot fully explain the development of NFPA or its associate morbidity, the likelihood of multiple genomic interactions intertwining to promote NFPA is probable.

The rs67307131 T > C polymorphism in the Pleckstrin homology-like domain, family B, member 1 (PHLDB1) was found in another study to be significantly associated with NFPA (odds ratio (OR) = 2.15, 95% confidence interval (CI) 1.44–3.20, *p* < 0.0002) in the Korean population [31]. The functional advantage of this polymorphism remains unclear; however, this data reveals a potential genetic marker of NFPA in a specific patient population.

**Figure 1:**
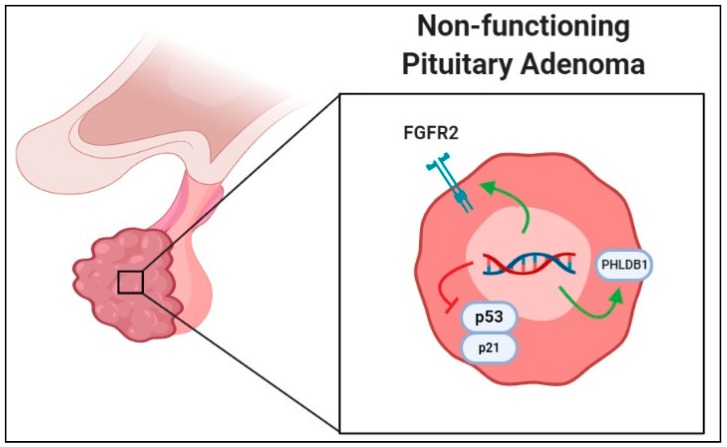
Graphical representation of single-nucleotide polymorphism associated with increased growth in nonfunctional pituitary adenomas (NFPAs) in the literature. Green arrows represent polymorphisms leading to increased gene product activity or transcription, while red line indicates that gene products are negatively affected to lead to increased NFPA growth. Abbreviations: FGFR2, fibroblast growth factor receptor 2; PHLDB1, Pleckstrin homology-like domain, family B, member 1.

Regardless of positive data of SNP correlation or involvement with NFPAs, there are studies reporting negative data as well on certain polymorphisms that are found to have no significant role in NFPA growth. One such study by Ruggeri et al. (2014) investigated SNPs in stimulatory Gs-protein and inhibiting Gi_2_ proteins (GNAS_1_ and GNAI_2_, respectively) as related to aberrant signaling and found that G-protein mutations are rare and not crucial in NFPA development [32]. Additionally, SNPs seemingly important in functional PAs may not have a role in tumorigenesis of NFPA, as Hu et al. reported when they found that polymorphisms of the *AIP* gene did not seem to confer any developmental advantage in NFPAs [33]. This is contrasted by the considerable amount of research linking *AIP* mutations with tumorigenesis of sporadic or familial functional pituitary tumors across various geographic and ethnic groups. Despite the negative results reported in the literature, a trend towards investigating the relationship between SNPs and NFPA formation can be demonstrated. Figure 1 is a graphical representation of the aforementioned SNPs.

### 2.5. SNPs in Functional Pituitary Adenomas

#### 2.5.1. SNPs in Prolactin-Secreting PAs (Prolactinomas)

PRL-secreting PAs, or prolactinomas, are the most common of all functional PAs, comprising of nearly 30%–50% of all secreting pituitary tumors [34]. These neoplasms arise from the lactotroph cells and—as their name suggests—produce PRL, which can then cause endocrine imbalance including amenorrhea, infertility, or galactorrhea [35]. Diagnosis of prolactinomas is based on peripheral PRL levels and cranial imaging, and once identified, first-line therapy is medical management with dopamine agonist agents, such as cabergoline or bromocriptine. These medications act by inhibiting PRL release and reducing tumor size, though side effects can reduce patient compliance. Due to first line medical management for prolactin-secreting PAs, it is important then to note that “normal” prolactinomas are not extensively studied. Thus, this represents a considerable bias because aggressive prolactinomas not amiable to medical management represent a small subset of all these tumors seen clinically.

Polymorphisms in growth factor receptors, tumor suppressors, and cell cycle regulators are associated with formation of prolactinomas, while SNPs in drug transporter genes have been reported to predict side effects of treatment with cabergoline. In a study by Ikeda H. (2006), the presence of a rs2228048 G > A SNP was found in the exon 4 sequence for transforming growth factor-beta receptor type II (TGF-beta RII), in 50% of patients with prolactinoma within the series [36]. Interestingly, decreased expression of TGF-beta RII, as found in knockout murine models, results in accelerated prolactinoma formation in mice, thus implicating this protein and associated pathway as tumor-suppressing in wild-type conditions [37]. Furthermore, proteins found downstream of the TGF-beta RII signaling pathway were found to be affected by polymorphisms to the SMAD3 gene in 29% of patients in the study cohort (rs1065080 C > T in exon 2), ultimately implicating aberrance in the TGF-beta RII pathway in prolactinoma development. In another study looking at the polymorphisms of cell surface receptor genes and formation of PAs, Peculis et al. (2016) found that in their cohort of 143 PAs (46 prolactinomas) and 354 case controls, the rs7131056 at the dopamine receptor D2 gene (*DRD2*) was associated with higher occurrence of extrasellar growth in patients with prolactinomas [38]. Thus, they concluded that the DRD2 polymorphism either contributes to faster growth of the adenoma or to reduced symptomatic presentation, allowing for the PA to become larger before detection.

Cell cycle regulator polymorphisms have also been studied in the context of prolactinoma formation. Cander et al. (2012) investigated the effects of the G870A gene polymorphism of cyclin D1 (*CCND1*) on the formation and behavioral features of prolactinomas [39]. The *CCND1* gene is a potent proto-oncogene that has been shown to be frequently altered in human tumors. With excessive expression of CCND1 protein, an increase occurs in cell cycle progression and increased proliferation is observed [40]. The authors reported that while no significant correlation exists between the G870A SNP and the biological behavior of prolactinomas, the frequency of the A allele at this position was higher in prolactinoma patients than in controls, which may ultimately suggest that *CCND1* G870A polymorphism may be an important factor in early stages of tumor formation. In a separate study by Simpson et al. (2001), polymorphisms of the *CCND1* gene were compared within a cohort of 294 pituitary adenomas (57 prolactinomas), and the incidence of A allele and the rate of A/A genotype was only significantly increased according to tumor grade in prolactinomas [41].

Given that medical management is first-line, polymorphisms that affect drug transport molecules are exceedingly relevant. The central side effects of dopamine agonists may be related to drug transport across the blood–brain barrier—the transporter being encoded by the *ABCB1* gene. In order to determine whether polymorphisms of *ABCB1* predict central side effects of cabergoline, Athanasoulia et al. (2012) performed a case control study involving 79 prolactinoma patients and studied four *ABCB1* SNPs (rs1045642, rs2032582, rs2032583, and rs2235015) [42]. Individuals with rs1045642 and rs2032582 SNPs in ABCB1 were found to have central side effects, namely fatigue, sleep disorder, and dizziness, when compared with the other two SNPs [42]. These results are valuable because use of SNPs to predict occurrence of central side effects of medical management for prolactinomas may be a conduit for personalized therapy for patients.

**Figure 2:**
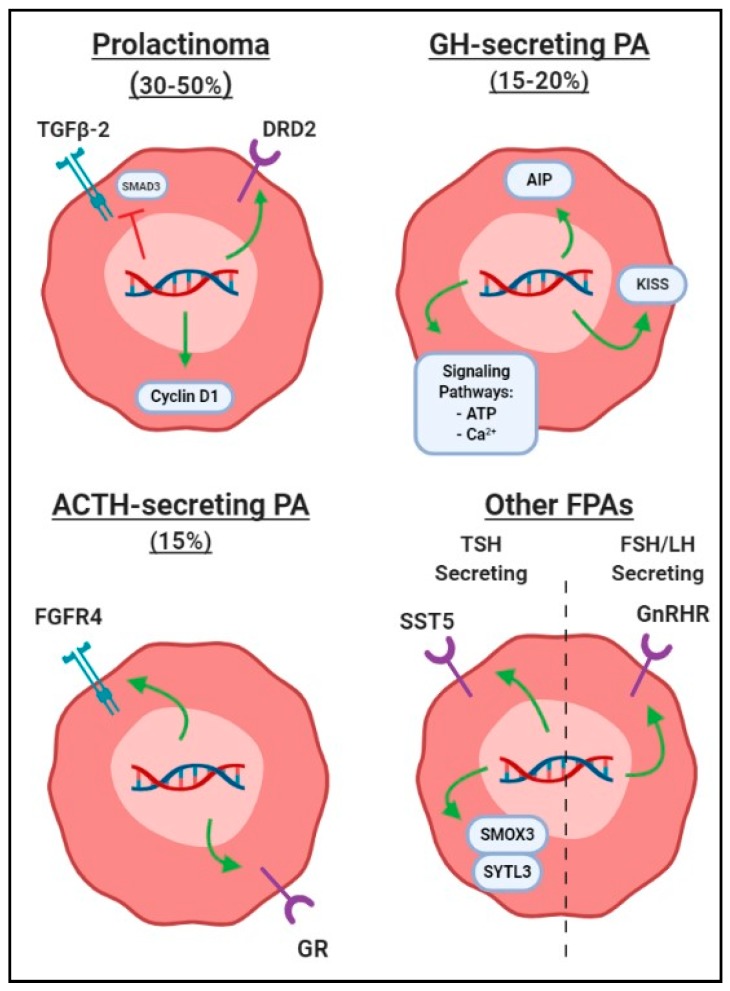
Graphic depiction of common single-nucleotide polymorphisms associated with formation of functional pituitary adenomas (FPAs), differentiated by hormone secretion subtype. Green arrows represent polymorphisms leading to increased gene product activity or transcription, while red line indicates that gene products are negatively affected to lead to increased FPA growth. Abbreviations: ACTH, adrenocorticotropin; FPA, functional pituitary adenoma; FSH, follicle-stimulating hormone; GH, growth hormone; LH, luteinizing hormone; PA, pituitary adenoma.

Figure 2 summarizes polymorphism products related to growth of prolactinomas, among other functional PAs to be discussed. Table 1 summarizes SNPs by different functional categories.

#### 2.5.2. SNPs in Growth Hormone-Secreting PAs

Growth hormone-secreting PAs (GHPAs), or somatotroph adenomas, are the second most common of the functional PAs and cause acromegaly as a result of hypersecretion of growth hormone [43]. As a result, acromegalics are predisposed to higher morbidities and earlier mortality secondary to cardiopulmonary or metabolic complications, so prompt diagnosis and treatment is imperative. Diagnosis is made from a combination of clinical history, blood screen for insulin growth factor-1 levels, and cranial imaging. Treatment modality first includes gross total resection if possible, followed by adjuvant medical management using somatostatin analogues, GH receptor antagonists, or dopamine agonists in cases of residual tumor [44].

Välimäki et al. (2015) set out to perform a whole-genome sequencing along with SNP array analysis of GHPAs on 12 fresh-frozen adenomas, all but one being negative for germline mutations [45]. This genome-wide analysis revealed around 129 somatic SNPs per tumor case, and further analysis of coding regions showed 2.3 SNPs per tumor. Despite no new recently mutated genes being detected, several SNPs in genes involved in adenosine ATP signaling and calcium channel signaling were found in multiple—pathways known to be involved in pituitary tumorigenesis [45]. This is of relevance in GHPAs given that cyclic AMP and Ca^2+^ signaling lead to increased cytosolic free calcium, which then propagates a release of GH [46,47]. In addition, ATP release induces increased intracellular calcium concentrations, which then promote GH secretion [48].

Polymorphisms in the fibroblast growth factor receptor 4 (*FGFR4*) have been shown to promote growth in GHPAs, as well as modulate the activity of somatostatin analogs. Tateno et al. (2011) found that the FGFR4-G388R polymorphism facilitated GHPA tumorigenesis via increased mitochondrial serine phosphorylation of STAT3 and Src activation, both increasing GHPA cell growth, driving increased cellular oxygen consumption, and disrupting GH feedback signaling [49]. This SNP carries a therapeutic relevance as well, as seen with a follow-up publication by Ezzat et al. (2017), in which they suggest that the somatostatin analog pasireotide—but not octreotide—is effective in decreasing oxygen consumption in FGFR4-G388R cells [50]. By comparison, the normal genotype FGFR4-G388 cells are equally targetable by both somatostatin analogs.

As previously described, the AIP gene is commonly associated with formation of functional pituitary tumors. Yarman et al. (2015) sought to determine whether *AIP* SNPs play a role in pathogenesis of familial and sporadic hormone-secreting pituitary adenomas and found that while there were no *AIP* mutations in their cohort, two exonic SNPs (rs641081 (Q228K) and rs4930195 (Q307R) were identified on the *AIP* locus [51]. Of these, the frequency of Q228K variants was higher in sporadic GHPAs than in case controls, indicating that *AIP* SNPs independently of full gene mutation may be related to formation of GHPAs. Another *AIP* polymorphism, rs2066853 identified in exon 10, was more frequently associated with acromegalic patients than healthy subjects and associated with increased disease aggressivity [52].

Additionally, SNPs in promoter regions of genes of interest can also be related to GHPA pathogenesis. Amorim et al. (2019) looked at promoter SNPs of a gene (*KISS1*) encoding for a protein, kisspeptin (KISS), to find a relationship between increased translation of KISS1 and GHPA formation [53]. KISS and its cognate receptor, KISSR, are broadly expressed at the pituitary level and increased KISS may stimulate GH release [54]. In Amorim and colleague’s study, 49 GHPAs and 167 healthy controls were assessed for *KISS1* c.−145delA (rs57802180) promoter polymorphism. The authors found that rs57802180 may be associated with incidence of GHPA but not with tumor progression.

Along with a concentration of evidence suggesting that the presence of certain SNPs confers an advantage to GHPA tumorigenesis, some studies have found that other SNPs may reduce the risk of developing acromegaly. A recent article from Gao et al. (2018) included 102 acromegalics and 143 control subjects to determine the effect of the rs2854744 A > C SNP at the −202 locus of insulin-like growth factor binding protein-3 (*IGFBP3*) and whether it constitutes a risk factor for acromegaly [55]. This study revealed that the C allele of rs2854744 is associated with reduced risk of acromegaly (odds ratio 0.594, 95% confidence interval 0.388–0.909). This correlation was more prominent in females in a Chinese population. Ultimately, these results suggested that *IGFBP3* may be involved in the acromegaly development. In a follow-up article, Gao and colleagues sought to evaluate the relationship between IGFBP3-202 A > C gene polymorphism and clinical features and surgery outcome in acromegalic patients [56]. They found that while polymorphisms in *IGFBP3* may not influence metabolic or GHPA characteristics in acromegalics, these polymorphisms may be associated with the hormone levels and surgery effects. There was also a trend that more acromegalics carrying the C allele needed additional treatment postoperatively (odds ratio 1.985, 95% confidence interval 0.983–4.008, *p* = 0.056) than A carriers.

#### 2.5.3. SNPs in Adrenocorticotropin-Secreting PAs

Adrenocorticotropin (ACTH)-secreting PAs, or corticotroph adenomas, represent around 15% of all functioning PAs and generally secrete ACTH leading to Cushing’s disease [34]. ACTH-secreting PAs appear to be monoclonal, suggesting that spontaneous somatic mutations are the primary pathogenic mechanism in the formation of these tumors [57,58]. This contrasts with corticotroph hyperplasia, which was found in one study to be polyclonal in nature [59]. Patients with ACTH-secreting PAs often present clinically with signs and symptoms of glucocorticoid excess, giving them the classic “cushingoid” appearance—weight gain, moon facies, abdominal striae, and buffalo hump [60]. Diagnosis is made using a combination of biochemical testing for ACTH levels, morning cortisol levels, and dexamethasone suppression testing, all of which can be used to differentiate primary ACTH-secreting PAs versus primary adrenal tumors. Cranial imaging then confirms the presence of a sellar mass. Treatment is primarily surgical with remission rates between 60%–90% [17].

While the evidence described above supports corticotrophic adenomas as being monoclonal in nature, making somatic mutations a likely etiology, several studies have revealed increased incidence of somatic polymorphisms in patients with corticotrophic PAs, suggesting that these polymorphisms may promote the growth of these adenomas to sufficient size to cause clinically diagnosable Cushing’s disease. For example, some studies have identified the importance of glucocorticoid receptor (GR) polymorphisms in tumorigenesis [61]. As corticotrophic adenomas are characterized by relative resistance to the negative feedback action of cortisol on ACTH secretion, it is reasonable to surmise that SNPs in *GR* genes may play some role in tumorigenesis, if not also tumor phenotype maintenance. Huizenga et al. (1998) were the first to describe that polymorphisms in the *GR* gene locus (chromosome 5) led to loss of heterozygosity in about 30% of a cohort of Cushing’s disease patients (as compared with 3.5% of control), suggesting a role for this *GR* gene polymorphism in ACTH-secreting tumors [62]. Upon further investigation of *GR* gene polymorphisms in ACTH-secreting tumors, Antonini et al. (2002) identified *GR* gene polymorphisms at codon 363 (N363S A > G) and at codon 766 (N766N T > C) in 17% and 11% of tumors, respectively [63]. While their results suggested that *GR* gene polymorphisms play little role in clinical presentation, tumor size, or surgical outcome, SNPs in the *GR* gene may confer a selective advantage to tumorigenesis in ACTH-secreting tumors.

The phenotypic features of ACTH-secreting PAs may also be related to FGF receptor 4 (*FGFR4*) polymorphic variants. A recent paper by Nakano-Tateno et al. found that expression of the minor FGFR4-R388 allele enhanced STAT3 serine phosphorylation driving cellular growth, which clinically manifested as silent corticotroph macroadenomas [64]. In contrast, expression of the major FGFR4-G388 allele is related to formation of hormonally active microadenomas. Furthermore, the FGFR4-G388 allele was found to be associated with reduced disease-free survival in another study by Brito and colleagues, in which the genotype of 76 study participants was collected and compared with clinical, hormonal, and pathological tumor data [65]. Within the same study, FGFR4 overexpression was found in 44% of ACTH-secreting tumors and was also associated with lower postoperative remission rate (*p* = 0.009).

#### 2.5.4. SNPs in Other Hormone-Secreting PAs

Less common types of functional PAs include gonadotropic (follicle-stimulating hormone (FSH) secreting and luteinizing hormone (LH) secreting) and thyrotropic (thyroid-stimulating hormone (TSH) producing) adenomas. Most gonadotropic adenomas though are silent, thus presenting with symptoms of mass effect, and will then be categorized based on histopathological findings. Unfortunately, the data found regarding nonfunctioning gonadotropic adenomas is relatively sparse. Kottler et al. (1998) found that functional gonadotropic adenomas were more likely to show a silent C to T transition at nucleotide 453 within the gonadotropin-releasing hormone receptor (*GnRHR*) gene than nonfunctioning gonadotropic adenomas [66]. This study was limited by a small patient sample, so larger studies would be warranted to fully understand the degree of influence imparted onto functional gonadotropic adenomas by *GnRHR* polymorphisms. TSH-secreting PAs are usually macroadenomas with extrasellar extension [67]. Treatment, as with the previously discussed PAs, revolves around correction of mass effect via surgical resection and amelioration of residual tumor and/or hormonal imbalances [68]. TSH-secreting PAs are not associated with genetic syndromes or conserved somatic mutations across tumors. A recent study combined mutational and SNP array analysis on eight TSH-secreting PAs. The authors found six candidate driver mutations, with an average of 1.5 somatic mutations per tumor (range 0–4) [69]. However, no mutations occurred in multiple patients. Two DNA variants were found in genes with an established role in tumorigenesis (*SMOX* and *SYTL3*), but the other four had unknown roles. In this study, an SNP array analysis was also performed, revealing frequent chromosomal regions of copy number gains, including recurrent gains at loci harboring four of the six genes for which mutations were identified. In another study of TSH-secreting PAs, Filopanti et al. (2004) found that polymorphisms in the somatostatin receptor type 5 gene (*SST5*) lead to loss of heterozygosity, which then leads to a more invasive phenotype in TSH-secreting PAs [70]. This suggests that SNPs may influence tumor aggression in TSH-secreting PAs, though further studies would be necessary to confirm this.

## 3. Future Directions

While the translational application of SNPs for the treatment, prognostic evaluation, and classification of PAs is still in its nascency, these applications have tremendous potential and warrant further investigation. Currently, there are significant limitations present in current studies that would need to be addressed prior to broad use of polymorphic data as the standard of care in PA management. Much of the literature pertaining to genomic polymorphisms in the context of functional and/or nonfunctional PA tumorigenesis is limited by small patient sample sizes from single centers. Thus, results are difficult to generalize. It is also to be determined whether individual polymorphisms are enough to drive tumorigenesis or if tumorigenesis requires a concert of multiple polymorphisms.

Once sufficiently large multi-center studies have been performed to validate the polymorphism studies in PAs to date and provide more mechanistic insight, future application of SNPs in PA patient care will likely involve multiple aspects. First, genetic testing for these polymorphisms could identify patients who, if harboring asymptomatic NFPAs, could be safely observed through serial imaging, or, alternatively, could identify patients with asymptomatic NFPAs for whom elective resection might be justified based on natural history linked to polymorphisms or patients with PAs that are more aggressive than average in terms of postsurgical recurrence rates. Other future lines of investigation related to polymorphisms and PAs include developing personalized medical treatments for NFPAs with particular polymorphisms.

## 4. Conclusions

While some somatic mutations underlie PA tumorigenesis, polymorphisms involving specific genes that regulate common cellular functions from tumor suppression to cell cycle and proliferation have been described in PAs. While NFPAs and functional PAs share polymorphisms of certain genes and pathways such as AIP, there are also distinct differences in polymorphisms between not only NFPAs and functional PAs, but also amongst the functional PA subtypes. Across multiple PA subtypes, some polymorphisms occur in genes whose functions could relate to tumorigenesis and others whose functions could relate to tumor progression or recurrence. While the translational relevance of SNPs in the formation of PAs is still in the early stages, the use of wide-scale genomic analysis to identify patients at risk for developing PAs may likely yield therapeutic benefit in the future. Larger, concentrated efforts to study SNPs in PAs will need to be executed first.

## Figures and Tables

**Table 1:** List of single-nucleotide polymorphisms by different functional categories and clinical relevance.

Functional Category	Gene	Tumor Type	SNP	Relevance
Tumor Suppressor	*p53*	NFPA	rs1042522 C > G	Increased cell proliferation and vascularity
	*TGF-B RII*	PRL	rs2228048 G > A	Accelerated prolactinoma formation
Growth Factor Receptor	*FGFR2*	NFPA	rs2981582	Increased activation of pro-mitogenic/survival downstream effectors
	*DRD2*	PRL	rs7131056	Associated with extrasellar prolactinoma growth
	*FGFR4*	GHPA	G388R	Increased cell proliferation, increase O_2_ consumption, and disruption of normal GH feedback response
	*FGFR4*	ACTH-PA	G388R	Increased cell proliferation
	*GR*	ACTH-PA	N363S A > G	Unclear, higher in ACTH-PA patients
			N766N T > C	Unclear, higher in ACTH-PA patients
	*GnRHR*	FSH/LSH	Nucl 453 C > T	Associated with functional gonadotropic adenomas > nonfunctional
Cell Cycle Regulator	*CCND1*	PRL-PA	G870A	Increased cell proliferation
Promoter	*KISS1*	GHPA	rs57802180	Increased KISS1 expression increases GH release
Drug-related	*ABCB1*	PRL-PA	rs1045642	Associated with central side effects in medically treated pts
			rs2032582	Associated with central side effects in medically treated pts
	*FGFR4*	GHPA	G388R	May modulate efficacy of somatostatin analog therapy
Miscellaneous	*PHCDB1*	NFPA	rs67307131 T > C	Unclear, possible genetic marker
	*AIP*	GHPA	rs641081 Q228K	Associated with acromegalic patients > health controls
			rs4930195 Q307R	Associated with acromegalic patients > health controls
			rs2066953	Associated with acromegalic patients > health controls
	*IGFBP3*	GHPA	A > C (unspec)	Unclear, possible association with need for postoperative medical therapy
	*MMP-1*	unspec	1G/1G genotype	More frequent in invasive vs. non-invasive PAs
	*MMP-9*	unspec	rs3918242 C > T	Higher MMP-9 observed in invasive vs. noninvasive PAs

Abbreviations: ACTH-PA, adrenocorticotropin-secreting pituitary adenoma; FSH, follicle-stimulating hormone; GH, growth hormone; GHPA, growth hormone-secreting pituitary adenoma; NFPA, nonfunctioning pituitary adenoma; PRL, prolactinoma; SNP, single-nucleotide polymorphism; unspec, unspecified.
